# Revealing the Role of Social Support on Cognitive Deficits in Fibromyalgia Syndrome

**DOI:** 10.1155/2022/3852746

**Published:** 2022-09-01

**Authors:** Carmen M. Galvez-Sánchez, Gustavo A. Reyes del Paso, Casandra I. Montoro

**Affiliations:** Department of Psychology, University of Jaén, Spain

## Abstract

Despite the relevance of cognitive deficits in fibromyalgia syndrome (FMS) and the attempts to elucidate the influence of the disorder symptoms in the cognitive decline reported by patients, no studies have explored the specific role of social support on cognition in FMS. Social support has been shown to be an essential modulator factor on cognitive performance in other diseases. Sixty-four women with FMS and 32 healthy women participated in the study and completed questionnaires pertaining to anxiety, depression, fatigue, insomnia, clinical pain, and social support, along with a neuropsychological battery assessing verbal memory, organization, strategic and planning abilities, self-regulation, processing speed, attention, and cognitive flexibility. Results showed that FMS patients exhibited lower values in all social support dimensions in comparison with healthy individuals, especially in the socializing dimension. Despite the lower social support observed in FMS, all social support dimensions showed a positive impact on verbal memory, organization and planning abilities, strategic planning, self-regulation, processing speed, attention, and cognitive flexibility in these patients. In fact, social support was associated with greater correct responses and processing speed and minor number of errors in all the neuropsychological battery tests. Socializing was the main predictor of organization and planning abilities, strategic planning, and self-regulation. In sum, results suggest that social support may be a key factor in buffering the cognitive decline observed in FMS. Designing psychoeducation programs and intervention programs directed not only to FMS patients but also relatives, health care workers, and the general population might be essential to improve the social support of FMS patients and positively impact on patient's cognitive status.

## 1. Introduction

Fibromyalgia syndrome (FMS) is a chronic pain disorder with a prevalence around 2-4% in the general population, being more frequent in women than in men [1]. FMS may be conceptualized as a widespread and persistent musculoskeletal pain, accompanied by several symptoms such as fatigue, insomnia, morning stiffness, depression, anxiety, and cognitive problems [1–3]. Cognitive impairments, which negatively impact on patient's life, frequently comprise problems in memory, attention, concentration, language, cognitive flexibility, and processing speed, along with reduced organization and planning abilities, among others, and are considered between the most disabling and worrisome symptoms of the disease [3–8].

Emotional aspects also play a relevant role in FMS. FMS has been associated with high negative affectivity [9–11], pain catastrophizing [12–16], alexithymia [17–19], self-esteem, and self-efficacy deficits [3, 20–23]. Negative affectivity (e.g., anxiety, depression, pain catastrophizing, and alexithymia) increases the intensity and severity of symptoms in FMS, worsening the quality of life of these patients [10, 12, 24, 25]. In addition, these emotional aspects have been associated with lessen cognitive performance in FMS [3, 5, 7, 18, 26–30], indicating the relevance of emotional aspects in cognitive deficit in FMS. In the same line, emotional factors are increasing its relevance due to the transdiagnostic perspective, which is showing greater scientific support and playing a crucial role in clinical management [3, 11, 31, 32]. Moreover, the transdiagnostic perspective seems to be also crucial for personalized behaviour management, which has shown to be essential for mood regulation as an alternative to medications [33].

Furthermore, other factor that can influence FMS symptoms is social support [34]. Social support may be conceptualized as resources provided to people in need by their social network and may be measured through the individual's perception of the degree to which interpersonal relationships can fulfil certain social support functions [35]. Social support is part of the social network function of each individual, generally related to the number and/or frequency of contacts with family members, relatives, friends, and colleagues [36]. Social support generally comprises several dimensions such as emotional, instrumental, appraisal (which implies information relevant to self-evaluation), and information, among others [34].

At this regard, some studies have reported a lack of social support in FMS patients [37, 38]. Although studies exploring the social support role in FMS symptoms are scarce, social support seems to contribute to improve mental and physical health in FMS patients [34]. In fact, the positive social interaction subcategory of social support has showed a negative association with the Fibromyalgia Impact (measured with the Fibromyalgia Impact Questionnaire) [34], depression state [34, 39], and alexithymia [40]. Besides, social support has been strongly related to anxiety, burnout, and severity of pain in FMS patients [39]. Similarly, Montoya et al. [41] reported that FMS patients perceived less general pain and thermal pain sensitivity as well as diminished brain activity elicited upon tactile stimulation of a tender point when the significant other was present in comparison with when the patients were alone, confirming the notion of social support as a factor explaining pain processing not only at the subjective behavioural level but also at the central nervous system level [41]. Similarly, a recent study confirmed the analgesic effects of social support, which was even observed without verbal or physical contact [42]. Partner empathy seems to reduce affective distress during pain exposure, decreasing pain sensitivity and promoting pain coping [42]. This is congruent with the proposed contribution of poor psychosocial functioning and unsatisfactory relationships in the genesis and maintenance of chronic pain [40].

However, the role of social support on FMS patient's cognition has not been studied until now. Previous studies in other populations confirm that the social support plays an important role involving in the maintenance or enhancement of mental health and cognitive functioning in elderly people [43–49], caregivers [50], and academic performance [51–53]. Therefore, based on these results, it might be hypothesized a similar effect of social support on cognitive performance in FMS patients.

Although the multifactorial nature of FMS has been stablished, research regarding family, work, and social support needs to be increased [54]. Considering the above-reviewed literature, the main objective of this research is to analyse, for the first time, the effect of social support on cognitive performance in FMS (specifically verbal memory, organization, strategic and planning abilities, self-regulation, processing speed, attention, and cognitive flexibility). Studies as the present one can contribute to development prevention and intervention programs aimed at improving the quality of life of FMS patients. To the best of our knowledge, this is the first study which assess the impact of social support in cognitive performance in FMS.

## 2. Materials and Methods

### 2.1. Participants

In total, 64 women with FMS, recruited from the AFIXA (Fibromyalgia Association of Jaén, Spain), participated in the study. All of them were examined by a rheumatologist and met the 1990 and 2010 American College of Rheumatology criteria for FMS [1, 2]. The control group comprised 32 healthy women. Given the main research objective of the study, analyses were restricted to the FMS group. The control group was only used for comparative purposes. Exclusion criteria for both study groups included the presence of metabolic abnormalities, neurological disorders, drug abuse, and severe somatic (e.g., cancer) or psychiatric (e.g., psychotic) diseases. Healthy individuals were further required not to suffer from any kind of acute or chronic pain. All participants were right handed.

### 2.2. Instruments and Measures

#### 2.2.1. Psychological Assessment

A semistructured interview was performed to obtain the patients' clinical history and sociodemographic data. The Structured Clinical Interview for Axis I Disorders of the Diagnostic and Statistical Manual for Mental Disorders (SCID) [55] was applied to assess the presence of possible mental disorders. Furthermore, the following self-report questionnaires were administered (values of Cronbach's *α* are reported from the available literature):

- State-Trait Anxiety Inventory (STAI) [56, 57]. This 20-item 4-point Likert scale's instrument allows for the assessment of current and habitual anxiety (e.g., state anxiety and trait anxiety, respectively; score range: 0-60). Cronbach's *α* = .93 for the state anxiety and .87 for trait anxiety [57]
- Beck Depression Inventory (BDI) [58, 59]. This 21-item scale was applied to assess depression (4-point Likert scales, scores range: 0-63). Cronbach's *α* = .95 [59]
- Fatigue Severity Scale (FSS) [60, 61]. This scale allows assessment of fatigue based on 9 items (7-point Likert scales, score range: 9-63). Cronbach's *α* = .88 [61]
- Oviedo Quality of Sleep Questionnaire (OQSQ) [62]. The insomnia subscale of this instrument, comprising of 9 items (5-point Likert scales, score range: 9-45), was used in the study. Cronbach's *α* of insomnia = .88 [62]
- McGill Pain Questionnaire (MPQ) [63, 64]. This 73-item instrument evaluates the different dimensions of pain. In the current research, the total pain experience (score range: 0-167) and current pain intensity (MPQ) were used. Cronbach's *α* of total pain = .74 [64]
- SS-B Social Support Scale [65, 66] is a 45-item (4-point Likert scales) questionnaire which allow to obtain information of five modes of supportive behaviours: emotional support, socializing, practical assistance, financial assistance, and advice/guidance. Moreover, this questionnaire is typically completed with respect to family and friends separately (family support and friends' support, respectively), providing information about the supportive behaviour available from relatives and friends. Cronbach's *α* = .82 [65]

#### 2.2.2. Cognitive Assessment

- Zoo Map Task (ZMT) from the Behavioural Assessment of the Dysexecutive Syndrome [67, 68] was used to evaluate the planning and organizational abilities. In this test, the participant has to plan a route to visit 6 of 12 possible locations in a zoo. The ZMT has two parts: (1) a more demanding open situation, in which little information is provided that would help to generate an appropriate plan, and (2) a situation that implies simply following a concrete, externally imposed strategy. Execution time and number of errors in each part in addition to the total number of correct responses were used as performance indices
- Verbal Learning Test (TAVEC) [69] assesses verbal memory function. At the beginning of the test, a list of 16 words (shopping list) is read to the participant five times (list A); the participant has to reproduce as many words as possible directly after each trial (immediate free recall). Immediately after, another list is read once (list B) and then has to be reproduced (interference control condition). Following a 20 min break, the words of list A have to be reproduced again (long-delay recall). Thereafter, a list of 44 words is read, which includes all words of list A, some words of list B, and further distractor words included in neither list A nor list B. The participant has to decide whether or not each of these words is part of list A (recognition). In the analysis, list A (immediate free recall), list B (interference control), short-term free memory, short-term guided memory or with semantic keys, long-term free memory, long-term guided memory or with semantic keys, and recognition correct responses were used as performance parameters. Guided memory refers to the trials in which words are reproduced according to semantic categories (e.g., fruits, clothes or tools).
- Revised Strategy Application Test (R-SAT) contains three paper-and-pencil activities, all of which include visual, motor, and linguistic abilities, specifically, figure tracing, sentence, assigned a weighted value, summed, divided by the total possible score for each category, and multiplied by 100. R-SAT allows to measure the strategic planning and self-regulation [70]. The task includes three simple activities, e.g., figure tracing, sentence copying, and object numbering. Activities are presented in two different stacks of 120 items each one. Items differ in terms of their size (large, small) and time requirements (brief, medium, long). A large item scores 0 points, and a small item scores 100 points, where participants are instructed to get as many points as possible. In addition, items in which a face is displayed have to be avoided. The items are intermixed; nevertheless, the number of brief items decreases progressively within both stacks. As the execution time of the task is restricted to 10 min, the most efficient strategy is to complete brief items instead of longer ones. Therefore, the predisposition to complete items in the presented sequence has to be overcome. R-SAT further provokes an unstructured environment in the laboratory in which environmental cues and internal habits oppose the most efficient strategy, thus reproducing the real-life situations. At the end of the task, participants are asked about the strategy which, according to their appraisal, was optimal to get the maximal number of points [70]. Participants also have to mark in a separate sheet when they think a minute has been spent (control marks) without using any watch. Performance was indexed by the number of correct answers (brief items), errors (long items and faces), and control marks
- Trail Making Test (TMT) [71, 72] evaluates processing speed, attention, and cognitive flexibility. The test, in which visual targets (numbers, letters) are presented on sheets of paper, includes the following tasks, all of which have to be executed as fast as possible: (1) visual scanning (cross out all number 3s on a page with different numbers), (2) number sequence (connect the numbers 1 to 16 in sequential order), (3) letter sequence (connect the letters A to P in alphabetic order), (4) switching (connect numbers and letters in alternating order, e.g., 1, A, 2, and B), and (5) motor speed (trace a predefined path). In addition to execution time, the following kinds of errors were recorded: For condition 1: (1) omissions (when the participant fails to mark any 3) and (2) commissions (when the person marks a letter or a number other than 3); for the rest of conditions (2, 3, and 4): (3) sequence (connection of correct item with an incorrect one), (4) set loss (connection of items of different categories) and (5) time out (exceeding the time limit of 250 s).

### 2.3. Procedure

The study was conducted in one session, divided in two parts conducted on the same day. During the first part, a clinical psychologist took the patients' clinical history, recorded sociodemographic data and medication use, and verified the inclusion and the exclusion criteria. Later, SCID interviews were carried out, and the questionnaires were fulfilled. In the second part, the neuropsychological battery was administered in the following order: ZMT, R-SAT, TAVEC (free recall), TMT, and TAVEC (second part). The tests were presented in this order to avoid the possible interference effect of the different cognitive domains, especially in verbal memory. Five breaking minutes was provided between each test. The study protocol was approved by the Ethics Committee for Human Research of the University of Jaén, and all participants provided written informed consent.

### 2.4. Statistical Analysis

In order to determine the optimal sample size based on expected effect sizes, the G∗Power 3.1.7 program was used [73]. Assuming an effect size of .75 and an alpha level of .05 and a beta error of 20% as a basis, a sample size from 21 participants per group appeared optimal. Comparisons between FMS patients and healthy individuals in clinical and demographic variables were performed using *F*-tests and *χ*^2^-tests. Group differences in cognitive performance were analysed by means of multivariate analysis of variance (MANOVA). Age, years of education, and body mass index were entered as covariates in this analysis (MANCOVA). A second MANCOVA was performed with the purpose to determine the possible role of emotional (i.e., state and trait anxiety and depression) and clinical variables (i.e., insomnia and fatigue) in cognitive performance. Effect sizes are indicated by adjusted eta squared (*n*_*p*_^2^). Associations between social support questionnaire dimension scores and neuropsychological test performance were evaluated in two steps, both restricted to the FMS group (*N* = 64). Firstly, at an exploratory level, Pearson correlations were computed. Secondly, multiple regression analyses were performed. Two blocks of variables were used as predictors in the analyses: (1) to control for the effects of age, body mass index, and years of education, these variables were entered simultaneously (enter method); (2) to determine social support predictive power for cognitive performance, the dimensions of the social support scales that showed significant correlations with the different neuropsychological parameters, in the exploratory analysis, were included (stepwise method) together with total and intensity of clinical pain (MPQ). The inclusion of clinical pain as a possible predictor lies in its relevance as one of the main explanatory mechanisms of cognitive deficits in FMS [7, 8, 26, 74, 75]. The SPSS software (version 22.0) was employed for data analysis (IBM Corporation, Armonk, NY).

## 3. Results and Discussion

Table 1 displays the sociodemographic and clinical data of both study groups. FMS patients displayed higher values for all clinical and emotional variables in comparison with healthy individuals (all ps < .0001). In addition, FMS patients displayed lower values for all social support variables compared to healthy individuals (all ps < .05, except financial assistance (SSB) (*p* = .050) and friends' support (SSB) (*p* = .363)).

### 3.1. Group Differences in Cognitive Performance

Table 2 shows neuropsychological test scores of FMS patients and healthy individuals and statistics of the univariate group comparisons. The MANOVA for the neuropsychological battery scores showed a multivariate group effect (*F* [26, 69] = 3.39, *p* < .0001, *n*_*p*_^2^ = .56). Moreover, this multivariate group effect (*F* [26, 66] = 2.96, *p* < .0001, *n*_*p*_^2^ = .54) remains significant in the MANCOVA (using as covariables the age, BMI, and years of education). Additionally, the second MANCOVA (including as covariable state and trait anxiety, depression, fatigue, and insomnia) showed that the multivariate group effect (*F* [26, 64] = 1.71, *p* = .042, *n*_*p*_^2^ = .41) remains significant. On purpose, also at the multivariate level, state anxiety (*F* [26, 64] = 1.41, *p* = .132, *n*_*p*_^2^ = .37) and insomnia (*F* [26, 64] = .91, *p* = .599, *n*_*p*_^2^ = .27) did not show any significant effect. By contrast, trait anxiety (*F* [26, 64] = 2.18, *p* = .006, *n*_*p*_^2^ = .47), depression (*F* [26, 64] = 3.77, *p* < .0001,  *n*_*p*_^2^ = 61), and fatigue (*F* [26, 64] = 1.90, *p* = .020, *n*_*p*_^2^ = .44) exhibited a significant multivariate effect.

### 3.2. Correlations between Social Support and Cognitive Performance in FMS Patients

Table 3 displays correlations between social support dimensions and cognitive performance in FMS patients.

All SSB dimensions were positively associated with organization and planning abilities and strategic planning and self-regulation (total correct responses of ZMT and correct responses: short items of R-SAT). In addition, emotional support (SSB) and friends' support (SSB) were positively associated with verbal memory (list A: immediate free recall of TAVEC). Emotional support (SSB) was also positively associated with verbal memory (short-term guided memory of TAVEC). Practical assistance (SSB), socializing (SSB), and friends' support (SSB) were positively associated with strategic planning and self-regulation (control marks of R-SAT).

All SSB dimensions were also negatively associated with errors in organization and planning abilities and strategic planning and self-regulation tasks (error versions 1 and 2 of ZMT and error long items of R-SAT). Moreover, emotional support (SSB), practical assistance (SSB), socializing (SSB), financial assistance (SSB), and advice/guidance (SSB) were negatively associated with errors in strategic planning and self-regulation (error face items of R-SAT). Emotional support (SSB) was also negatively associated with processing time, attention, and cognitive flexibility (execution time 2: number sequence of TMT). Family support (SSB) was negatively associated with the time spending in organization and planning abilities (execution time version 2: ZMT). Practical assistance (SSB), socializing (SSB), financial assistance (SSB), and family support (SSB) were negatively associated with processing speed, attention, and cognitive flexibility (omission errors of TMT). All dimensions, except for family support (SSB), were negatively associated with processing speed, attention, and cognitive flexibility (sequence errors of TMT).

### 3.3. Results of Multiple Regression Analysis

Table 4 shows the significant results of the multiple regression analyses for the prediction of performance parameters, after controlling for the effects of age, years of education, and BMI. At this regard, related to the first model, socializing (SSB) was the main predictor of organization and planning abilities and strategic planning and self-regulation (total correct responses of ZMT and R-SAT and errors of both versions in the ZMT test). In addition, current pain intensity (MPQ) was the main predictor of organization and planning abilities; processing speed, attention, and cognitive flexibility; and verbal memory (execution time in the version 2 of ZMT, execution time 2: number sequence of TMT, and short-term guided memory of TAVEC). Moreover, financial assistance (SSB) was the main predictor of strategic planning and self-regulation (error long items and face items of R-SAT), whereas friends' support (SSB) was the principal predictor of strategic planning and self-regulation, verbal memory, and processing speed, attention, and cognitive flexibility (control marks of R-SAT, list A: immediate free recall of TAVEC and sequence errors of TMT). And family support (SSB) was the main predictor of processing speed, attention, and cognitive flexibility (omission errors of TMT).

Regarding the second models, current pain intensity (MPQ) was the main predictor of organization and planning abilities (total correct responses of ZMT and error version 2 of ZMT). Furthermore, advice/guidance (SSB) was the main predictor of errors in organization and planning abilities (error version 2 ZMT). Besides, practical assistance (SSB) was the main predictor of strategic planning and self-regulation and verbal memory (correct responses: short items of R-SAT, error face items of R-SAT, and list A: immediate free recall of TAVEC). Emotional support (SSB) was the main predictor of processing speed, attention, and cognitive flexibility (execution time 2: number sequence of TMT).

## 4. Discussion

The main objective of the current research was to analyse the effect of social support on the cognitive performance in FMS (including verbal memory, organization, strategic and planning abilities, self-regulation, processing speed, attention, and cognitive flexibility). The present results reaffirm the higher values of clinical and emotional symptoms (e.g., clinical pain, insomnia, fatigue, depression, and anxiety) in FMS compared to healthy individuals [3, 4, 6–8, 18]. Furthermore, the cognitive impairments in FMS (especially in verbal memory, organization and planning abilities, strategic planning, self-regulation, processing speed, attention, and cognitive flexibility) are confirmed in line with the available scientific evidence [3–8].

Results of the second MANCOVA further reveal that group differences in cognitive performance seem to be independent of emotional and clinical symptoms [76, 77]. At this regard, Roldán-Tapia et al. [76] pointed out that cognitive impairment in FMS patients could not be explained by the collateral effects of such pathologies, because cognitive profiles were different and appeared from the onset of the disease notion also supported by the research of Simos et al. [77].

Refer to social support, FMS patients displayed lower values for all social support variables compared to healthy individuals [37, 38], especially in the socializing area. Nevertheless, there was no any significant difference in financial assistance and friends' support between both groups. Although more studies are required to firm clear conclusions, some research has suggested that FMS patients seem to be more likely to include their physicians as intimate members of their social networks and less willing to take initiative in meeting new people than patients with rheumatoid arthritis (RA) [78]. This may explain the lower observed social support values associated with the socializing sphere in FMS patients. This finding also reinforces the significant role of health professionals in FMS [79]. Another explanation might relapse in the fact that FMS patients usually perceive little social support at work due to the lack of social knowledge and awareness on the disease [80]. This lack of social support reduces personal relationships at work. This is influenced by the misunderstanding about the lower effectiveness of FMS patients at work [80]. At the same time, the problems in the professional field and the need to stop working in some cases because of the illness symptoms might worsen the social isolation. It is well known that staying in the workplace prevents FMS patients from social isolation and reduces the negative impact of the disease on their quality of life [81]. Therefore, it is necessary to design programs to increase the training and sensitization of relatives and friends of FMS patients, as well as of health providers and general population [79].

Related to social support and cognitive performance in FMS patients, all studied SBB dimensions (e.g., emotional support, practical assistance, socializing, financial assistance, advice/guidance, family support, and friends' support) were positively related to a better cognitive performance (higher levels of correct responses) in the organization and planning abilities (ZMT) and strategic planning and self-regulation tasks (R-SAT). Similarly, practical assistance, socializing, and friends' support (SSB) were positively linked to higher strategic planning and self-regulation performance (R-SAT). Moreover, emotional support and friends' support were positively associated with a better performance in the verbal memory domain (TAVEC).

In addition, the social support was not only positively related with the number of correct responses of neuropsychological test but also was associated with a reduction of the processing speed. In fact, family support was negatively associated with execution time version 2 of ZMT, which measures organization and planning abilities, whereas emotional support was also negatively linked to execution time 2 (number sequence) of TMT which assesses processing speed, attention, and cognitive flexibility.

Regarding the number of errors, social support also might be involved in the reduction of these one. In this sense, practical assistance, socializing, financial assistance, advice/guidance, family support, and friends' support were negatively associated with errors in organization and planning abilities tasks and strategic planning and self-regulation tasks (errors of both versions in ZMT and error long items of R-SAT, respectively). Additionally, emotional support, practical assistance, socializing, financial assistance, and advice/guidance were negatively related to error face items of R-SAT, which measure strategic planning and self-regulation performance. And all dimensions of the social support scale were negatively associated with different errors of TMT, which assesses processing speed, attention, and cognitive flexibility.

Moreover, socializing was the main predictor of organization and planning abilities and strategic planning and self-regulation (total correct responses of ZMT and R-SAT and errors of both versions in the ZMT test). One possible explanation can be the positive effect of social support in pain processing at the subjective behavioural level and at the central nervous system level of FMS patients [41]. In addition, an analgesic effect of social support has been reported in healthy population, even without verbal or physical contact [42]. Similarly, it is possible that socializing might have an analgesic effect on pain (one of the main explanatory factors of cognitive impairments in FMS [8, 74], which indirectly improve the cognitive performance of patients). Otherwise, this analgesic effect of social support in pain processing might be also favouring the cognitive processing areas. It is well known that pain is an attention-demanding stimulus that recruits brain areas also relevant for cognitive processing [8, 74, 82]. It would be interesting to assess the possible mediating role of social support in the relation between pain and cognitive performance in FMS.

Our research reveals the positive effect of social support especially in verbal memory, organization and planning abilities, strategic planning, self-regulation, processing speed, attention, and cognitive flexibility. Results are in line with previous studies suggesting a positive association between social support and health outcomes in FMS patients [34, 39–41].

Additionally, emotional support showed to be the social support's variable that account for the majority of associations with the cognitive parameters, suggesting, among all SBB dimensions, a special role of it in FMS cognition. This finding is according to previous studies that pointed out that social support, and particularly emotional support, was associated with decreases in health care use within a primary care setting [83]. Unfortunately, although the previous mentioned research is very meaningful, no cognitive aspects were evaluated, being this study the first one on this issue. Therefore, the role of social support on cognitive performance in FMS can be considered a research gap that needs to be overcome to better understand the disease and provide FMS patients a more holistic and personalized treatment. In addition, emotional support would need also to be promoted based on the transdiagnostic perspective which insist on the relevance of emotional and social aspect of the illnesses apart from treat on the disease symptoms [31, 32]. Furthermore, based on previous findings [33], it might be hypothesized the relevance of improving social support, especially emotional support, in order to personalized FMS treatment.

Nonetheless, despite the role of social support in FMS needs to continue being studied, our results may be explained based on previous research in other populations, which point out that social support is a significant factor involved in the maintenance or enhancement of mental health and cognitive functioning in general (e.g., elderly people [43–49], caregivers [50], and academic performance [51–53, 84]). In this regard, it has been noted that social relationships, especially social activities and networks, have a protective effect against greater cognitive decline in older adults [85, 86]. Cotton et al. [87] explored the neural correlates of social support in older adults and reported the existence of a gray matter network related to social support (including prefrontal, hippocampal, amygdala, cingulate, and thalamic regions), which was in turn associated with memory and executive function. Cacioppo and Hawkley [88] further showed that this gray matter network associated with tangible social support was composed by regions previously linked to memory, executive function, aging, and dementia. Authors concluded that more longitudinal research of the interrelationships between social support, brain structure, and cognition was needed and advised of the importance of strengthen social support as a new path toward improving cognition in aging [88]. Moreover, research and interventions in this field would help to better understand the contribution of poor psychosocial functioning (e.g., social support) and unsatisfactory relationships in the origin and maintenance of chronic pain [40], especially in FMS.

Likewise, social isolation (e.g., loneliness) has been proposed as a risk factor for and may contribute to poorer overall cognitive performance, faster cognitive decline, poorer executive functioning, and higher negativity and depressive cognition [88]. Loneliness has exhibited a mediating role between social isolation and subjective cognitive impairment in older people [89–92], including older immigrants [89]. The association between loneliness and subjective cognitive deficits has been well-stablished [93, 94]. On purpose, social support, indeed, is known to play a critical role in the detection and treatment of mild cognitive impairment [95–97].

Loneliness has been proposed as a risk factor of dementias, especially Alzheimer disease [96–99]. In general, higher levels of social engagement have been related to greater levels of cognition across the lifespan, association that seems to be more significant in populations at risk of cognitive impairment [100]. The research in other populations confirmed the protective role of social support in cognitive performance, although the exact nature of this association remains unclear [46, 85, 86, 89, 93, 94, 100]. Previous research also highlights the need of promoting psychosocial interventions and related public health strategies to enhance neurocognitive health, increasing specific forms of social support [94, 100–102], such as supportive listening [102]. In fact, social support group interventions in people with dementia and mild cognitive impairment exhibited psychological benefits, specifically, a reduction of depression and an improvement of the quality of life and the self-esteem [103]. Considering the prevalence and negative impact of cognitive deficits in FMS, it is worth keep exploring the effect of social support at this regard.

The main limitation of our study was its cross-sectional design, which does not allow for the establishment of causal associations. Longitudinal studies are required to better understand the association between social support and cognitive impairment in FMS. It also would be interesting to compare FMS patients with other clinical samples (e.g., RA patients), to determine if the observed association between social support and better cognitive performance is or not specific of FMS but can be extended to other pain conditions. Strengths of the study included the novelty and clinical relevance of the results, the statistical control of sociodemographic variables, and the determination of the sample through the G∗Power program to ensure the statistical power of the analysis. Moreover, social support in FMS has been evaluated conforming the recommendation of not only to consider the amount of support reported by the patients but also the type of support they received [40].

Due to the relevance of cognitive deficits in FMS [3–8, 104], a routine screening for cognitive impairments in these patients should be included in both diagnosis and treatment [105]. Moreover, taking the biopsychosocial perspective into account, it must be also mandatory to explore and promote the social support network of these patients in order to prevent the worsening of cognitive performance and subsequent health-related quality of life. It is necessary to continue researching the mechanism through the social support benefit the cognitive performance in chronic pain patients, including FMS, and confirming the protective role of social support in this kind of disorders. It might be a promising future line of research and clinical practice to improve the quality of life of these patients.

Furthermore, the design of psychoeducation programs and intervention programs to improve the social support of FMS cannot only positively influence on patient's status but also in the health system as proposed by previous studies [106, 107]. In fact, some researches showed that one's social network was positively linked to health status and negatively related to health care use, reducing prescriptions, laboratory tests, and visits to a nurse, nurse practitioner, and/or physicians' assistant [107]. Nonetheless, it is important to highlight that for stablishing an effective social support network for FMS patients, it is necesary to provide relatives and friends with diseasse information for a better understanding. Therefore, it is important to also involve them in the psychoeducation programs [108]. At this regard, Kool et al. [109] analysed if social support and invalidation (lack of understanding and discounting by others) were differently related to physical and mental health. They studied 1455 patients with different chronic pain diseases, such as FMS, RA, ankylosing spondylitis, and osteoarthritis [109]. Their results confirmed that both invalidation and social support were linked to patients' mental health, but only invalidation was significantly related to patients' physical health, suggesting the relevance to include social support and invalidation in programs to improve health of patients with rheumatic diseases [109]. Besides, the therapeutic adherence in some FMS programs seems to be associated with some factors such as lack of motivation and lack of social support, among others [110]; therefore, enhancing the social support of these patients might have a positive impact in their treatments.

## 5. Conclusions

This is the first study exploring the influence of social support in cognitive performance of FMS patients. FMS displayed lower values in all social support variables compared to healthy individuals, especially in the socializing area, which was the main predictor of organization and planning abilities, and strategic planning and self-regulation of these patients (total correct responses of ZMT and R-SAT and errors of both versions in the ZMT test). All dimensions of social support exhibited a positive impact on verbal memory, organization and planning abilities, strategic planning, self-regulation, processing speed, attention, and cognitive flexibility. Social support dimensions not only positively impact on the number of correct responses and processing speed of neuropsychological test but also seem to reduce the number of errors. Improving the social networks of FMS patients might help to ameliorate their health status and cognitive performance while simultaneously reducing health care utilization. It is vital to involve not only FMS patients but also their relatives and friends along with health care workers and general population, in order to improve the FMS knowledge and sensitize people to the importance of social support in this disease.

## Figures and Tables

**Table 1:** Sociodemographic and clinical variables and questionnaire scores in FMS patients (*N* = 64) and healthy individuals (*N* = 32) groups (M ± SD or number and %). Statistics of group comparisons are also included (*F*-test or *χ*^2^-test).

	FMS patients	Healthy individuals	*F* or *χ*^2^	*p*	*n*_*p*_^2^
Age	52.73 ± 7.89	51.13 ± 6.61	.99	.323	.01
Body mass index (BMI)	28.00 ± 5.03	26.06 ± 3.05	3.39	.069	.04
Education (years)	10.28 ± 4.30	12.00 ± 4.34	4.02	.050	.04
Antidepressant use (%)	53 (%)	5 (%)	40.27	<.0001	.65
Anxiolytic use (%)	50 (%)	5 (%)	34.06	<.0001	.60
Analgesic use (%)	53 (%)	3 (%)	47.34	<.0001	.70
Opiate use (%)	33 (%)	0 (0%)	25.14	<.0001	.51
Trait anxiety (STAI)	50.67 ± 10.61	26.13 ± 15.95	80.66	<.0001	.46
State anxiety (STAI)	23.20 ± 4.25	15.56 ± 11.65	21.91	<.0001	.19
Depression (BDI)	43.52 ± 13.56	14.28 ± 17.36	81.92	<.0001	.47
Fatigue (FSS)	52.45 ± 9.99	25.66 ± 16.10	100.51	<.0001	.52
Insomnia (OQSQ)	39.09 ± 11.10	14.16 ± 9.62	117.34	<.0001	.56
Total pain (MPQ)	79.23 ± 36.18	19.03 ± 25.23	71.11	<.0001	.43
Current pain intensity (MPQ)	3.64 ± 1.20	1.28 ± .73	104.13	<.0001	.53
Emotional support (SSB)	44.44 ± 36.00	64.47 ± 53.02	4.77	.031	.05
Practical assistance (SSB)	35.14 ± 22.50	49.19 ± 39.38	4.95	.029	.05
Socializing (SSB)	30.80 ± 18.70	43.03 ± 34.11	5.17	.025	.05
Financial assistance (SSB)	36.16 ± 24.74	49.38 ± 39.67	4.01	.050	.04
Advice/guidance (SSB)	50.34 ± 32.00	73.72 ± 58.52	6.42	.013	.06
Family support (SSB)	102.61 ± 61.95	174.59 ± 161.81	9.86	.002	.10
Friends' support (SSB)	78.16 ± 72.00	94.06 ± 95.22	.84	.363	.01

Note: STAI: State-Trait Anxiety Inventory; BDI: Beck Depression Inventory; FSS: Fatigue Severity Scale; OQSQ: Oviedo Quality of Sleep Questionnaire; MPQ: McGill Pain Questionnaire; SSB: SS-B Social Support Scale.

**Table 2:** Mean (±SD) of neuropsychological test scores of FMS patients (*N* = 64) and healthy individuals (*N* = 32) and statistics of the univariate group comparisons.

		FMS patients	Healthy individuals	*F* [4, 66]	*p*	*n*_*p*_^2^
ZMT	Total correct responses	12.13 ± 2.84	13.38 ± 2.49	3.43	.067	.04
	Execution time version 1	239.33 ± 127.90	179.06 ± 82.45	3.87	.052	.04
	Execution time version 2	159.34 ± 107.42	90.13 ± 50.32	7.91	.006	.08
	Error version 1	2.89 ± 1.96	1.94 ± 1.16	4.90	.029	.05
	Error version 2	.88 ± 1.06	2.22 ± 10.56	.80	.373	.01

TAVEC	List A (immediate free recall)	37.50 ± 9.98	46.31 ± 10.52	11.85	.001	.12
	List B (interference control)	3.53 ± 1.89	4.53 ± 2.00	3.82	.054	.04
	Short-term free memory	7.91 ± 3.43	10.44 ± 2.97	9.25	.003	.09
	Short-term guided memory	9.59 ± 2.73	10.84 ± 3.03	2.30	.133	.03
	Long-term free memory	8.80 ± 2.82	10.53 ± 3.20	4.96	.028	.05
	Long-term guided memory	9.19 ± 2.86	11.28 ± 2.84	8.10	.005	.08
	Recognition correct responses	9.28 ± 6.06	15.16 ± 1.51	24.95	<.0001	.22

R-SAT	Correct responses (short items)	44.22 ± 9.11	50.34 ± 7.50	13.52	<.0001	.13
	Error long items	4.95 ± 7.41	2.03 ± 2.40	4.97	.028	.05
	Error face items	1.61 ± 2.10	.44 ± .91	8.31	.005	.08
	Control marks	4.84 ± 2.26	6.41 ± 2.28	10.00	.002	.10

TMT	Execution time 1 (visual scanning)	80.03 ± 108.79	41.88 ± 13.31	3.42	.068	.04
	Execution time 2 (number sequence)	98.55 ± 63.96	60.31 ± 25.71	7.42	.008	.08
	Execution time 3 (letter sequence)	105.34 ± 70.65	66.91 ± 30.18	5.61	.020	.06
	Execution time 4 (switching)	231.05 ± 130.98	119.16 ± 51.34	18.24	<.0001	.17
	Execution time 5 (motor speed)	140.91 ± 58.38	98.09 ± 49.73	7.77	.006	.08
	Omission errors	.22 ± .68	.06 ± .25	1.50	.224	.02
	Commission errors	.03 ± .18	.00 ± .00	1.76	.188	.02
	Sequence errors	2.28 ± 3.09	.91 ± 1.51	4.25	.042	.05
	Set loss errors	3.05 ± 5.65	.31 ± .69	6.53	.012	.07
	Time out errors	10.91 ± 10.80	.78 ± 2.06	22.05	<.0001	.20

Note: ZMT: Zoo Map Test; TAVEC: Verbal Learning Test; R-SAT: Revised Strategy Application Test; TMT: Trail Making Test. All execution times are indicated in s. ^∗^*p* < .05. ^∗∗^*p* < .01.

**Table 3:** Correlations between social support measured by SSB and cognitive performance in FMS patients (*N* = 64).

		Emotional support (SSB)	Practical assistance (SSB)	Socializing (SSB)	Financial assistance (SSB)	Advice/guidance (SSB)	Family support (SSB)	Friends' support (SSB)
ZMT	Total correct responses	.367^∗∗^	.387^∗∗^	.421^∗∗^	.396^∗∗^	.368^∗∗^	.360^∗∗^	.282^∗^
	Execution time version 1	.050	-.009	.046	.039	.063	.012	.118
	Execution time version 2	-.124	-.203	-.192	-.181	-.157	-.257^∗^	-.047
	Error version 1	-.385^∗∗^	-.419^∗∗^	-.438^∗∗^	-.434^∗∗^	-.371^∗∗^	-.326^∗∗^	-.364^∗∗^
	Error version 2	-.373^∗∗^	-.385^∗∗^	-.425^∗∗^	-.395^∗∗^	-.377^∗∗^	-.331^∗∗^	-.299^∗^

TAVEC	List A (immediate free recall)	.274^∗^	.101	.143	.129	.158	-.023	.269^∗^
	List B (interference control)	.138	.079	.068	.087	.083	.045	.071
	Short-term free memory	.223	.138	.163	.162	.131	.094	.203
	Short-term guided memory	.247^∗^	.150	.155	.181	.107	.016	.163
	Long-term free memory	.186	.189	.197	.194	.136	.049	.232
	Long-term guided memory	.162	.068	.111	.090	.027	-.064	.124
	Recognition correct responses	.134	.118	.094	.137	.159	.202	.095

R-SAT	Correct responses (short items)	.401^∗∗^	.404^∗∗^	.460^∗∗^	.411^∗∗^	.419^∗∗^	.312^∗^	.370^∗∗^
	Error long items	-.362^∗∗^	-.343^∗∗^	-.376^∗∗^	-.391^∗∗^	-.366^∗∗^	-.312^∗^	-.307^∗^
	Error face items	-.348^∗∗^	-.318^∗^	-.344^∗∗^	-.384^∗∗^	-.338^∗∗^	-.241	-.235
	Control marks	.094	.249^∗^	.252^∗^	.237	.225	.139	.258^∗^

TMT	Execution time 1 (visual scanning)	.003	.190	.217	.149	.072	.023	.124
	Execution time 2 (number sequence)	-.257^∗^	-.217	-.224	-.223	-.189	-.188	-.131
	Execution time 3 (letter sequence)	-.230	-.165	-.171	-.182	-.153	-.110	-.141
	Execution time 4 (switching)	-.220	-.226	-.233	-.200	-.204	-.156	-.139
	Execution time 5 (motor speed)	-.067	-.166	-.130	-.113	-.126	-.166	-.066
	Omission errors	-.190	-.262^∗^	-.251^∗^	-.252^∗^	-.204	-.356^∗∗^	-.120
	Commission errors	-.098	-.122	-.129	-.118	-.115	.029	-.197
	Sequence errors	-.254^∗^	-.262^∗^	-.306^∗^	-.293^∗^	-.270^∗^	-.116	-.308^∗^
	Set loss errors	-.174	-.175	-.201	-.217	-.186	-.169	-.122
	Time out errors	-.134	-.227	-.232	-.169	-.177	-.194	-.104

Note: ZMT: Zoo Map Test; TAVEC: Verbal Learning Test; R-SAT: Revised Strategy Application Test; TMT: Trail Making Test. ^∗^*p* < .05. ^∗∗^*p* < .01.

**Table 4:** Significant results of the second block (step-wise method) of the multiple regression analysis for the prediction of neuropsychological test scores by clinical pain and social support variables in FMS patients (*N* = 64).

		M	Predictors	*β*	Δ*r*^2^	*t*	*p*
ZMT	Total correct responses	1	Socializing (SSB)	.43	.18	4.20	<.001
		2	Socializing (SSB)	.43	.08	4.52	<.001
			Current pain intensity (MPQ)	-.30		-3.06	.003
	Execution time version 2	1	Current pain intensity (MPQ)	.30	.09	2.65	.010
	Error version 1	1	Socializing (SSB)	-.40	.15	-3.77	<.001
		2	Socializing (SSB)	-1.37	.05	-3.07	.003
			Advice/guidance (SSB)	1.00		2.24	.029
	Error version 2	1	Socializing (SSB)	-.39	.15	-3.50	.001
		2	Socializing (SSB)	-.39	.07	-3.68	.001
			Current pain intensity (MPQ)	.28		2.49	.015

TAVEC	List A (immediate free recall)	1	Friends' support (SSB)	.29	.08	2.53	.014
		2	Friends' support (SSB)	.74	.09	3.79	<.001
			Practical assistance (SSB)	-.55		-2.79	.007
	Short-term guided memory	1	Current pain intensity (MPQ)	-.34	.11	-2.89	.005

R-SAT	Correct responses (short items)	1	Socializing (SSB)	.47	.22	4.38	<.001
		2	Socializing (SSB)	1.44	.05	2.97	.004
			Practical assistance (SSB)	-.99		-2.05	.045
	Errors (long items)	1	Financial assistance (SSB)	-.38	.14	-3.15	.003
	Errors (face items)	1	Financial assistance (SSB)	-.39	.15	-3.21	.002
		2	Financial assistance (SSB)	-1.67	.08	-3.10	.003
			Practical assistance (SSB)	1.31		2.43	.018
	Control marks	1	Friends' support (SSB)	.26	.07	2.09	.041

TMT	Execution time 2 (number sequence)	1	Current pain intensity (MPQ)	.34	.10	3.21	.002
		2	Current pain intensity (MPQ)	.36	.05	3.53	.001
			Emotional support (SSB)	-.23		-2.23	.030
	Sequence errors	1	Friends' support (SSB)	-.31	.10	-2.57	.013
	Omissions errors	1	Family support (SSB)	-.36	.12	-2.89	.005

Note: Model (M), standardized *β*, change in *r*^2^ (Δ*r*^2^), *t*, and *p* are indicated. Results of the first block, which served to control for the effects of age, education, and BMI, are not reported. ZMT: Zoo Map Test; TAVEC: Verbal Learning Test; R-SAT: Revised Strategy Application Test; TMT: Trail Making Test.

## Data Availability

The data presented in this study are available on request from the corresponding author.
